# Clazakizumab in late antibody-mediated rejection: study protocol of a randomized controlled pilot trial

**DOI:** 10.1186/s13063-018-3158-6

**Published:** 2019-01-11

**Authors:** Farsad Eskandary, Michael Dürr, Klemens Budde, Konstantin Doberer, Roman Reindl-Schwaighofer, Johannes Waiser, Markus Wahrmann, Heinz Regele, Andreas Spittler, Nils Lachmann, Christa Firbas, Jakob Mühlbacher, Gregor Bond, Philipp F. Halloran, Edward Chong, Bernd Jilma, Georg A. Böhmig

**Affiliations:** 1 Division of Nephrology and Dialysis, Department of Medicine III, Medical University of Vienna, Währinger Gürtel 18-20, A-1090 Vienna, Austria; 2 Department of Nephrology, Charité University Medicine Berlin, Berlin, Germany; 3 Department of Clinical Pathology, Medical University of Vienna, Vienna, Austria; 4 Core Facility Flow Cytometry, Medical University of Vienna, Vienna, Austria; 5 Centre for Tumor Medicine, H&I Laboratory, Charité University Medicine Berlin, Berlin, Germany; 6 Department of Clinical Pharmacology, Medical University of Vienna, Währinger Gürtel 18-20, A-1090 Vienna, Austria; 7 Department of Surgery, Medical University of Vienna, Vienna, Austria; 8 Alberta Transplant Applied Genomics Centre, Faculty of Medicine & Dentistry, #250 Heritage Medical Research Centre, University of Alberta, Edmonton, AB Canada; 9 Vitaeris Inc., Vancouver, Canada

**Keywords:** Antibody-mediated rejection, Clazakizumab, Donor-specific antibody, Interleukin-6, Kidney transplantation, Monoclonal antibody

## Abstract

**Background:**

Late antibody-mediated rejection (ABMR) triggered by donor-specific antibodies (DSA) is a cardinal cause of kidney allograft dysfunction and loss. Diagnostic criteria for this rejection type are well established, but effective treatment remains a major challenge. Recent randomized controlled trials (RCT) have failed to demonstrate the efficacy of widely used therapies, such as rituximab plus intravenous immunoglobulin or proteasome inhibition (bortezomib), reinforcing a great need for new therapeutic concepts. One promising target in this context may be interleukin-6 (IL-6), a pleiotropic cytokine known to play an important role in inflammation and adaptive immunity.

**Methods:**

This investigator-driven RCT was designed to assess the safety and efficacy of clazakizumab, a genetically engineered humanized monoclonal antibody directed against IL-6. The study will include 20 DSA-positive kidney allograft recipients diagnosed with ABMR ≥ 365 days after transplantation. Participants will be recruited at two study sites in Austria and Germany (Medical University of Vienna; Charité University Medicine Berlin). First, patients will enter a three-month double-blind RCT (1,1 randomization, stratification according to ABMR phenotype and study site) and will receive either clazakizumab (subcutaneous administration of 25 mg in monthly intervals) or placebo. In a second open-label part of the trial (months 4–12), all patients will receive clazakizumab at 25 mg every month. The primary endpoint is safety and tolerability. Secondary endpoints are the pharmacokinetics and pharmacodynamics of clazakizumab, its effect on drug metabolism in the liver, DSA characteristics, morphological ABMR lesions and molecular gene expression patterns in three- and 12-month protocol biopsies, serum/urinary biomarkers of inflammation and endothelial activation/injury, Torque Teno viral load as a measure of overall immunosuppression, kidney function, urinary protein excretion, as well as transplant and patient survival.

**Discussion:**

Currently, there is no treatment proven to be effective in halting the progression of late ABMR. Based on the hypothesis that antagonizing the effects of IL-6 improves the outcome of DSA-positive late ABMR by counteracting DSA-triggered inflammation and B cell/plasma cell-driven alloimmunity, we suggest that our trial has the potential to provide proof of concept of a novel treatment of this type of rejection.

**Trial registration:**

ClinicalTrials.gov, NCT03444103. Registered on 23 February 2018 (retrospective registration).

**Electronic supplementary material:**

The online version of this article (10.1186/s13063-018-3158-6) contains supplementary material, which is available to authorized users.

## Background

Accumulating evidence has shown that antibody-mediated rejection (ABMR) is a leading cause of kidney allograft dysfunction and failure in the long-term [1–3]. The diagnosis of ABMR is based on the detection of donor-specific antibodies (DSA) and characteristic acute and/or chronic morphological lesions in the microvasculature and, in some cases, detection of complement split product C4d along peritubular capillaries [4]. In addition, ABMR was shown to associate with specific gene expression patterns [5] and molecular criteria have now been included in recent updates of the Banff classification of renal allograft pathology [4]. While a continuous diagnostic refinement has helped define the role of this rejection type as a major trigger of chronic allograft injury, treatment of late and/or chronic ABMR still represents a major challenge in clinical practice. Our current knowledge is mainly based on the results of uncontrolled observational studies evaluating a variety of therapeutic concepts including modifications in maintenance immunosuppression [6–8], administration of high-dose intravenous immunoglobulin and/or CD20 antibody rituximab [9–12], proteasome inhibition using bortezomib [13], or blockade of complement [14, 15]. The results of recent systematic randomized controlled trials (RCT) performed in late/chronic ABMR are disappointing as they failed to demonstrate efficacy of specific interventions, in particular, rituximab and intravenous immunoglobulin [16] and bortezomib [17].

The pleiotropic pro-inflammatory cytokine interleukin-6 (IL-6), which is known to play a pivotal role not only as a trigger of acute phase responses, but also in the activation and development of B cells and antibody production, may be a promising target for ABMR treatment [18]. Monoclonal antibodies against IL-6 and the IL-6 receptor (IL-6R) have been proven to be effective in the treatment of various autoimmune diseases, including rheumatoid arthritis [19–21]. More recently, targeting IL-6/IL-6R has gained interest also in the context of organ transplantation. In an uncontrolled study by Choi et al. [22], the anti-IL6R monoclonal antibody tocilizumab was evaluated as treatment of chronic ABMR unresponsive to intravenous immunoglobulin, rituximab, and/or plasmapheresis in a cohort of 36 kidney transplant recipients. The results of this study are promising, as they showed a beneficial safety profile, a reduction in DSA levels, and stabilization of kidney function at two years after treatment initiation. An interesting result was a marked reduction in the extent of microcirculation inflammation in follow-up biopsies [22].

Direct binding and blockade of IL-6 might be an attractive alternative. Clazakizumab (formerly ALD518 or BMS945429; Vitaeris Inc., Vancouver, Canada) is a genetically engineered high affinity humanized monoclonal antibody (IgG1), which binds to IL-6 and prevents its interaction and signaling via IL-6R. This antibody is the most potent and longest acting agent in the IL-6/IL-6R blocking category, is free of antibody-dependent cellular cytotoxicity as well as complement-dependent cytotoxicity and does not crosslink any surface receptors. In addition, its mode of action may have the advantage that, in contrast to IL-6R blockade, it may not include a rebound effect due to accumulated IL-6.

Clazakizumab has been evaluated extensively in patients with arthritis [20, 23–25], but has not yet been approved for any condition. The half-life of clazakizumab is about 30 days (after subcutaneous administration), and monthly subcutaneous injections allow for sustained IL-6 blockade. Its safety has so far been established in > 1000 subjects, many of them treated within phase 2 studies performed in rheumatoid arthritis or psoriatic arthritis [20, 23–25]. To date, no studies with clazakizumab have been conducted in patients with ABMR after renal transplantation and its safety profile in transplant recipients on maintenance immunosuppression—usually a combination of two or three different compounds—is so far unknown. A potential advantage of clazakizumab over IL-6R blockade (tocilizumab) in terms of efficacy and safety profile remains speculative.

Here, we describe the protocol of a 12-month pilot trial, which has primarily been designed to evaluate the safety and tolerability of clazakizumab in a cohort of 20 kidney transplant recipients diagnosed with late ABMR. The design of this study, which will include early and late follow-up protocol biopsies to decipher the effects on microcirculation inflammation, gene expression profiles, and features of chronic transplant injury, will also allow for a preliminary efficacy assessment (including information on effect size and variability), and thus can be expected to provide a valuable basis for future trials performed in large populations of transplant patients.

## Methods/Design

### Study design and trial flow

This prospective bi-centre study (Medical University Vienna and Charité University Medicine Berlin) is an investigator-initiated phase 2 pilot trial designed to assess the safety, tolerability, and efficacy (preliminary assessment) of the humanized anti-IL-6 monoclonal antibody clazakizumab in late ABMR. The sponsor of this non-commercial trial is the Medical University of Vienna. Apart from study design, the sponsor will carry out the trial (in collaboration with research partners), being responsible for all its scientific, ethical, regulatory, and legal aspects. The funder (Vitaeris Inc., Vancouver, Canada) has set funding conditions and will provide external funding.

We hypothesize that the administration of clazakizumab in kidney transplant recipients on top of baseline immunosuppression will have an acceptable safety profile, as earlier reported for patients with autoimmune diseases. Moreover, we hypothesize that repeated administration of clazakizumab will counteract tissue inflammation and injury in ongoing ABMR, in particular, inflammation in the microcirculation, human leukocyte antigen (HLA)-specific B cell alloresponse, and alloantibody-triggered chronic graft injury.

**Fig. 1:**
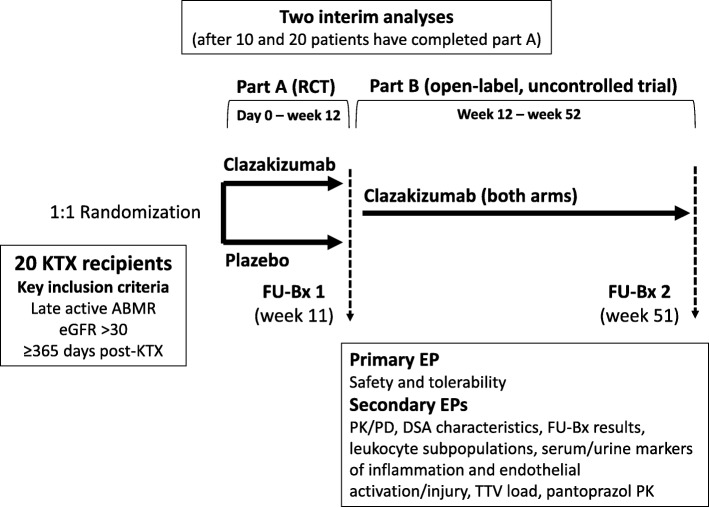
Study *flow chart*. DSA donor-specific antibody, eGFR estimated glomerular filtration rate, FU-Bx follow-up biopsy, KTX kidney transplantation, PD pharmacodynamics, PK pharmacokinetics, TTV Torque Teno virus

**Fig. 2:**
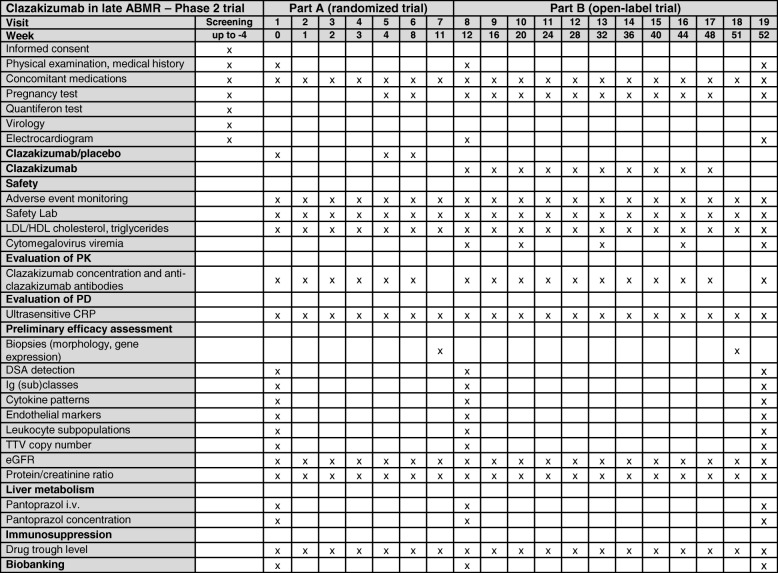
Schedule of events. CRP C-reactive protein, DSA donor-specific antibody, eGFR estimated glomerular filtration rate, HDL high density lipoprotein, Ig immunoglobulin, LDL low density lipoprotein, PD pharmacodynamics, PK pharmacokinetics, TTV Torque Teno virus

A flowchart of the trial is shown in Fig. 1. A schedule of events is provided in Fig. 2, and a signed SPIRIT 2013 checklist is attached as a supplementary file (Additional file 1). The study consists of two subsequent sub-parts. In a first part (part A), participants will be randomized to receive either clazakizumab (25 mg subcutaneous injection) or placebo for a period of 12 weeks (administration of clazakizumab/placebo at day 0 and after four and eight weeks). After 11 weeks, patients will be subjected to a first follow-up transplant biopsy. At week 12, part A will be completed and the randomization sequence will be unblinded for a first analysis of data. Primary goals of this part of the trial are to assess the safety and tolerability of a short course of treatment. Moreover, part A will allow for a first preliminary assessment of the impact of clazakizumab on ABMR-associated inflammation detected in peripheral blood and in the rejecting organ allograft. After week 12, all study patients will enter an open-label part of the study (part B) and will receive 25 mg clazakizumab in four-weekly intervals until the end-of-study visit after 52 weeks. After 51 weeks, patients will be subjected to a second protocol biopsy. Major goals of part B are to evaluate the safety and tolerability of a prolonged period of treatment with clazakizumab and the long-term impact of this antibody on the evolution of ABMR, rejection-associated biomarkers and kidney allograft function and survival over a period of 12 months.

Although the study was not designed to investigate any rebound effect after stopping treatment with clazakizumab, after the final study visit, all study participants will be monitored for any sign of worsening of renal transplant function in regular follow-up visits every four weeks for five months after the last dose (5 × half-life) in outpatient clinics.

We expect completion of patient recruitment after 18 months and finalization of the trial after 30 months.

### Participants

We will include kidney transplant recipients with circulating anti-HLA DSA and biopsy features of ABMR in an indication biopsy performed for a positive post-transplant DSA result and/or slow deterioration of allograft function and/or proteinuria. Other key inclusion criteria are a functioning graft at ≥ 365 days post-transplantation and an estimated glomerular filtration rate (eGFR) > 30 mL/min/1.73 m^2^. This eGFR threshold has been chosen to avoid inclusion of transplants with a high degree of irreversible chronic damage (for patients with very advanced graft injury a sustainable treatment benefit can no longer be expected). Inclusion and exclusion criteria are listed in Table 1.

**Table 1:** Inclusion and exclusion criteria

Inclusion criteria	1. Voluntary written informed consent
	2. Age > 18 years
	3. Functioning kidney allograft after ≥ 365 days after transplantation
	4. eGFR > 30 mL/min/1.73 m^2^
	5. Slow deterioration of graft function or proteinuria
	6. Detection of HLA class I and/or II DSA (preformed and/or de novo)
	7. Active or chronic/active ABMR (± C4d in peritubular capillaries)
	8. Molecular ABMR score ≥ 0.2
Exclusion criteria	1. Patients actively participating in another clinical trial
	2. Age ≤ 18 years
	3. Female participants are pregnant or lactating
	4. Index biopsy results:
	T-cell-mediated rejection classified Banff grade ≥ I
	De novo or recurrent severe thrombotic microangiopathy
	Polyoma virus nephropathy
	De novo or recurrent glomerulonephritis
	5. Acute rejection treatment < 3 months before screening
	6. Acute deterioration of graft function (> 25% eGFR decline within 1–3 months)
	7. Nephrotic range proteinuria > 3500 mg/g protein/creatinine ratio
	8. Active viral, bacterial, or fungal infection precluding intensified immunosuppression
	9. Active malignant disease precluding intensified immunosuppressive therapy
	10. Patients with evidence of malignant disease, or malignancies diagnosed within the previous 5 years (with the exception of local basal or squamous cell carcinoma of the skin or carcinoma in situ of the cervix uteri that has been excised and cured)
	11. Abnormal liver function tests (ALT, AST, bilirubin > 1.5× ULN)
	12. Other significant liver disease
	13. Latent or active tuberculosis (positive QuantiFERON-TB-Gold test, Chest X-ray)
	14. Administration of a live vaccine within 6 weeks of screening
	15. Neutropenia (< 1 G/L) or thrombocytopenia (< 100 G/L)
	16. History of gastrointestinal perforation, diverticulitis, or inflammatory bowel disease
	17. Allergy against proton pump inhibitors
	16. History of alcohol or illicit substance abuse
	17. Serious medical or psychiatric illness likely to interfere with study participation
	18. Prisoners or individuals who are involuntarily incarcerated
	19. Individuals who depended on the sponsor, the study site, or the investigators
	20. Patients with known hypersensitivity to any constituent of the product
	21. Patients with history of or currently active primary or secondary immunodeficiency

*ABMR* antibody-mediated rejection, *ALT* alanine aminotransferase, *AST* aspartate aminotransferase, *DSA* donor-specific antibody, *eGFR* estimated glomerular filtration rate, *HLA* human leukocyte antigen, *ULN* upper limit of normal

### Randomization procedure

For part A, patients will be randomized 1:1 to one of the two study arms (clazakizumab versus placebo) using a web-based randomization platform (https://www.meduniwien.ac.at/randomizer). Randomization will be stratified by study site and according to ABMR categories (active ABMR versus chronic/active ABMR) to ensure a balance of patients with these two histological types between the two arms.

### Sample size calculation

For this pilot study, no exact sample size estimation has been performed, because the effect size is unknown (there is no prior information to base a sample size on). The primary endpoint will be safety and tolerability. A preliminary assessment of efficacy outcomes in 20 kidney transplant recipients with late ABMR will provide first data on the effect of clazakizumab on clinical, morphological, immunological, and molecular endpoints. The respective results (e.g. comparison between clazakizumab and placebo with respect to microcirculation inflammation and molecular rejection scores), including an assessment of variability, can be expected to provide a valuable foundation of the design of future trials.

### Interventions

Clazakizumab or placebo will be administered as single subcutaneous injection of 1 mL each by blinded qualified study personnel. Clazakizumab will be supplied in single-dose vials (25 mg/mL) for injection and provided by Vitaeris Inc. The placebo medication will be administered with normal saline for injection and will be provided by the investigator. In part A of the trial, clazakizumab or placebo will be administered in four-weekly doses as single SC injection on day 0 and at weeks 4 and 8. During part B, all patients will receive four-weekly doses of clazakizumab starting at week 12, then at weeks 16, 20, 24, 28, 32, 36, 40, 44, and 48 (end-of study visit at week 52).

The following medications will be prohibited during the study: rituximab; eculizumab; proteasome inhibitors; intravenous immunoglobulin; plasma exchange or immunoadsorption; and other investigational drugs/treatments including commercially available anti-IL-6/IL-6R monoclonal antibodies such as tocilizumab. The following concomitant medications will be permitted during the study: calcineurin inhibitors (tacrolimus or cyclosporine A); mammalian target of rapamycin (mTOR) inhibitor (everolimus or rapamycin); mycophenolate mofetil (MMF)/mycophenolate sodium; and low dose corticosteroids (prednisolone ≤ 5 mg/day).

### Baseline immunosuppression

Upon diagnosis of late ABMR, all recipients (both arms in part A) on therapy with a calcineurin inhibitors or an mTOR inhibitor, without azathioprine or MMF/mycophenolic acid, will receive MMF (initially 2 × 500 mg per day; stepwise increase to 2 × 1000 mg per day if tolerated) to avoid under-immunosuppression. Our protocol does not allow for MMF doses > 2000 mg per day. There is a lack of solid data on the optimal dosing of MMF in ABMR; a concern in this respect may be that higher MMF doses could markedly increase the hematologic toxicity of IL-6 blockade (e.g. leukopenia). Tacrolimus will be adjusted to achieve target trough levels in the range of 5–10 ng/mL, CyA to 80–120 ng/mL. Recipients that had earlier been weaned off steroids will receive low dose prednisolone (5 mg/day). For each participant, the type of immunosuppression and its modifications during the study period will be documented.

### Blinding and unblinding

Randomization (part A) will be conducted at the two study sites by non-blinded pharmacists, who are also responsible for the preparation of clazakizumab and placebo (0.9% saline). To maintain and guarantee blinding and to avoid potential unblinding of group allocation, preparations of real drug and placebo will be identical in color, appearance, and smell. The participating investigators, medical interacting staff, and the study participants will be blinded to group allocation; the clinical team will not be able to access the secure allocation list (restricted password-protected access) until the last patient has completed part A. Premature unblinding may be necessary in cases of medical emergencies, serious medical conditions, where participants cannot be treated adequately unless the medical staff knows the allocated treatment conditions, or reports of suspected unexpected serious adverse events (SAEs). Unblinding can, if necessary, be requested by the data and safety monitoring board (DSMB).

### Outcome parameters

Primary and secondary outcome measures are detailed in Table 2. A schedule of events is provided in Fig. 2. Primary outcome measures are the safety and tolerability of clazakizumab, evaluated throughout the study period (19 visits until EOS visit at 52 weeks, day 0, weeks 1, 2, 3, 4, 8, 11, 12, 16, 20, 24, 28, 32, 36, 40, 44, 48, 51, and 52). According to the International Conference on Harmonization (ICH) statistical principles, safety concerns the medical risk to the participants, assessed by laboratory tests, vital signs, and AEs, while tolerability describes the degree to which overt AEs can be tolerated by the individual (as reflected by the rate of dropouts due to a lack of tolerability). Secondary endpoints are the pharmacokinetics (PK; clazakizumab concentration) and pharmacodynamics (PD) of clazakizumab, as assessed by serial measurements of C-reactive protein (CRP). Suppression of this hepatocyte-derived acute phase protein was earlier shown to be a valuable surrogate marker of anti-IL-6 efficacy [26]. For preliminary efficacy assessment, blood-derived biomarkers of ABMR (DSA characteristics, cytokines, and markers of endothelial activation/injury, patterns of peripheral blood leukocyte subpopulations) will be assessed at day 0 and after 12 and 52 weeks. In addition, we will study IL-6/IL-6R transcripts (before study initiation and at weeks 12 and 52), to clarify whether and to what extent neutralization of IL-6 modulates gene expression. The overall immunosuppressive effect of clazakizumab will be evaluated by monitoring of TTV load in plasma.

**Table 2:** Study endpoints

Primary outcome	Safety and tolerability (every visit)
Secondary outcomes	Every visit
	PK/PD of clazakizumab (antibody levels, CRP suppression)
	Kidney function (eGFR)
	Urinary protein excretion (protein/creatinine ratio)
	Biopsy-proven acute rejection necessitating rejection treatment
	Graft loss, death
	Day 0, week 12, and week 52
	PK of pantoprazole
	Cytokines and markers of endothelial activation/injury (serum, urine)
	Effect on leukocyte subsets in peripheral blood
	Effect on IL-6 and IL-6R gene expression in peripheral blood cells
	DSA characteristics
	DSA-MFI
	Number of DSA
	Broadness of sensitization (virtual panel-reactive antibody levels)
	Ig classes (IgG, IgA, IgM) and IgG subclasses (IgG1, 2, 3, 4)
	TTV load
	Week 11 and week 51
	Protocol biopsy results
	ABMR category
	Microcirculation inflammation (glomerulitis, peritubular capillaritis)
	Transplant glomerulopathy and interstitial fibrosis/tubular atrophy
	Molecular ABMR score
	Archetype analysis of gene expression profiles

*ABMR* antibody-mediated rejection, *CRP* C-reactive protein, *DSA* donor-specific antibody, *eGFR* estimated glomerular filtration rate, *HLA* human leukocyte antigen, *Ig* immunoglobulin, *MFI* mean fluorescence intensity, *PD* pharmacodynamics, *PK* pharmacokinetics, *TTV* Torque Teno virus, *ULN* upper limit of normal

**Table 3:** Summary of AEs reported for clazakizumab at 25 mg - phase 2 trials in arthritis

Disease entity	Psoriatic arthritis		Rheumatoid arthritis	
	Mease et al., 2016 [25]		Weinblatt et al., 2015 [20]	
Treatment	Placebo ± MTX	Clazakizumab ± MTX	Placebo + MTX	Clazakizumab + MTX
Patients (n)	41	41	61	59
Deaths (%)	0	0	0	0
Gastrointestinal perforation (%)	0	0	0	0
Malignancies (%)	0	0	0	0
SAEs (%)	4.9	4.9	3.3	8.5
Discontinuation (SAEs) (%)	4.9	0	0	0
AEs (%)	65.9	73.2	60.7	84.7
Discontinuation (AEs) (%)	7.3	2.4	0	0
Infections (%)	48.8	36.6	Total infection rate not reported	
Liver parameters (%)				
ALT > 1–3 × ULN	24.2	52.6	21.6	46
ALT > 3–5 × ULN	0	5.3	2	3.7
ALT > 5–8 × ULN	2.0	0	0	3.7
AST > 1–3 × ULN	13.5	50	14.8	40
AST > 3–5 × ULN	0	0	0	3.6
AST > 5–8 × ULN	0	2.6	0	0
Total bilirubin (%)				
> 1.0–1.5 × ULN	0	10.8	0	8.8
> 1.5–2.0 × ULN	0	5.4	0	1.8
> 2.0–3.0 × ULN	0	2.7	0	0
Cases of Hy’s law	0	0	0	0
LDL cholesterin	No details (lipids elevated)		Increase from < 130 to ≥ 130 mg/dL	
			28.3	61.9
Neutrophil counts	Mean decrease by about 2 G/L			
0.5–1.0 × 10^9^/L (%)	no details		0	1.8
1.0–1.5 × 10^9^/L (%)	no details		3.8	14.3
Anti-clazakizumab antibodies (%)	–	4.9	–	5.1
Injection site reaction (%)	–	9.8	–	13.6

*AE* adverse event, *ALT* alanine aminotransferase, *AST* aspartate aminotransferase, *SAEs* severe adverse event, *ULN* upper limit of normal

The study will include two protocol biopsies, an early biopsy after 11 weeks (before the end of part A) to primarily dissect the effect of IL-6 blockade on microcirculation inflammation in direct comparison to placebo, and a late biopsy after 51 weeks (before the end of part B) to assess the effect of clazakizumab on the progression of chronic injury (extent of transplant glomerulopathy, interstitial fibrosis, and tubular atrophy). The two biopsies will each be performed shortly (one week) before the end of the respective study parts, to avoid interference with detailed pharmacokinetic analyses scheduled for weeks 12 and 52, respectively. All biopsies will be evaluated for ABMR-typical gene expression patterns (ATAGC, Alberta University, Edmonton; major read-out: molecular ABMR score). In addition, kidney function, protein excretion, graft failure, and death will be evaluated over the whole study period. Finally, we will assess the impact of IL-6 blockade (as compared to placebo) on the PK of pantoprazole as a probe drug of cytochrome (CYP)-dependent liver metabolism. Pantoprazole was chosen, because single doses are well tolerated, and CYP2C19 activity is sensitive to phenoconversion during inflammation [27]. At day 0 and weeks 12 and 52, pantoprazole will be administered at a dosage of 20 mg intravenously and drug levels will be determined every hour over a period of 6 h. In patients on continuous proton pump inhibitor therapy, oral treatment will be paused for three days before PK testing and restarted one day thereafter.

### Safety evaluation and reporting of adverse events

In any clinical situation, including emergencies, adequate medical care will be provided. Safety evaluation will include a careful monitoring of all adverse events (AEs), including SAEs. There are no data available on the safety and tolerability of clazakizumab in transplant populations. From previous studies, however, results obtained in > 1000 clazakizumab-treated patients/participants are available for the assessment of safety patterns. Table 3 provides a summary of AEs reported for clazakizumab at a dose of 25 mg/month in two representative phase 2 trials performed in patients with arthritis.

### Interim analyses

This study will be monitored by an independent DSMB to assess the safety and data quality. To detect major differences between groups in terms of safety aspects in a timely manner, the board members will be instructed to perform interim analyses after 10 and 20 patients have finalized part A of the study. The DSMB will analyze recorded AEs and safety lab results in relation to the randomization sequence.

The DSMB may consider stopping the trial if the overall pattern of related SAEs or changes in safety lab results strongly support a major safety signal. Part A: if six participants experience related (definitely and possibly) SAEs (common toxicity criteria > Grade 3 or severe/medically significant) and/or substantially elevated levels of liver parameters (alanine aminotransferase [ALT], aspartate aminotransferase [AST], and/or bilirubin > 3× upper limit of normal [ULN]) or neutropenia (< 0.5 G/L), the DSMB will unblind the safety results. If five or all participants are in the clazakizumab group, then the study will be stopped. Similarly, if six related SAEs and/or substantial abnormalities in liver enzymes or neutrophil counts occur in the same system organ class, the DSMB will unblind these SAEs and if five or all SAEs are in the clazakizumab group, then the study will be stopped. Part B (all participants on clazakizumab treatment): if ≥ 10 individuals experience drug-related SAEs or ≥ 10 related SAEs occur in the same system organ class, then the trial will be stopped (the total number of related SAEs should also include the related SAEs occurring in the clazakizumab-treated individuals during part A).

For this pilot trial, exact statistical definitions of criteria for premature study termination are not defined.

### Quality control and quality assurance

Monitoring procedures will include predefined regular visits at the two study sites. The investigators will grant access to all source documents including case report forms and other protocol-related documents. Participant confidentiality will be maintained in agreement with local regulations. The designated monitor will contact and visit the investigator regularly and will be allowed to have access to all source documents needed to verify the entries in the case report forms and other protocol-related documents provided that participant confidentiality is maintained in agreement with local regulations. It will be the monitor’s responsibility to inspect the case report form at regular intervals according to the monitoring plan throughout the study, to verify the adherence to the protocol and the completeness, consistency, and accuracy of entered data. The monitoring standards require full verification for the presence of informed consent, adherence to the inclusion/exclusion criteria, documentation of AE and the recording of the main efficacy, safety, and tolerability endpoints.

### Methodology

#### Kidney function

eGFR will be assessed using the Chronic Kidney Disease Epidemiology Collaboration (CKD-EPI) equation (mL/min/1.73m^2^). Protein excretion will be documented as protein/creatinine ratio in spot urine (mg/g).

#### Transplant biopsies

Index and follow-up biopsies will be performed using ultrasound-guided percutaneous techniques (1–2 cores per biopsy, 16-gauge needle). After biopsy, patients will be monitored closely for 5–8 h for any complications (serial blood pressure measurements, monitoring for hematuria, hemoglobin check 4 h after biopsy). Histomorphology will be evaluated on paraffin-embedded sections applying standard methodology. For immunohistochemical C4d staining, we will use a polyclonal anti-C4d antibody (BI-RC4D, Biomedica, Vienna, Austria) and minimal immunohistochemical staining (C4d Banff score ≥ 1) along peritubular capillaries will be considered positive. Biopsies will also be evaluated by electron microscopy for detection of microcirculation injury. Morphological results will be evaluated by experienced renal transplant pathologists blinded for treatment allocation. Furthermore, biopsies will be analyzed using a thoroughly validated molecular method (MMDx™), as proposed by the Banff scheme [4]. For each biopsy, a 3-mm portion of one core will be placed immediately in RNAlater, stored at – 20 °C and shipped at ambient temperature or dry ice to the Alberta Transplant Applied Genomics Centre (ATAGC, University of Alberta, Edmonton, AB, Canada) for gene array analysis. Molecular scores based on machine-learning derived lesion-based classifiers related to rejection (ABMRpm, TCMRt, all Rejection) will be generated using the Edmonton reference set of > 1200 biopsies. Moreover, gene expression patterns will be evaluated using unbiased archetype analysis [28]. For classification of ABMR, all biopsy results will be analyzed in the context of the molecular results. ABMR categories and morphological single lesions will be defined and scored following recent updates of the Banff classification [4].

#### HLA antibody detection

Sera will be heat-inactivated to preclude complement-dependent in vitro artifacts (prozone phenomenon) and subjected to single-antigen flow-bead testing (LABscreen Single Antigen assays; One Lambda, Canoga Park, CA, USA). Test results will be documented as mean fluorescence intensities; a mean fluorescence intensity (MFI) > 1000 will be considered as positive. Donor specificity will be defined according to low- or high-resolution donor and recipient HLA typing results. Virtual panel-reactive antibody levels will be calculated using specific software tools (http://www.eurotransplant.org/cms/).

#### Cytokines and markers of endothelial activation/injury

Sera collected at weeks 0, 12, and 52 will be analyzed for IL-6, soluble IL-6R, and markers reflecting inflammation or endothelial activation and injury using bead arrays on a Luminex platform or enzyme-linked immunosorbent assay technology. In parallel, urine samples will be tested using the same methodology. For Luminex-based analysis of serum and urine biomarkers we will use Human ProcartaPlex Simplex Immunoassays following the manufacturer’s protocol (Thermo Fisher Scientific, Waltham, MA, USA). Tests will be performed on a Luminex 200 instrument. Bead panels will include combinations of different markers that potentially reflect the extent of inflammation and injury, including chemokines (e.g. CXCL9 and CXCL10) and endothelial markers (e.g. VCAM-1 and E-selectin), respectively. Urinary results will be normalized to urinary creatinine concentrations.

#### Leukocyte subpopulations

The underlying mechanisms of ABMR, especially the role of peripheral T- and B-cell subsets are not fully clarified. Thus, prospective immune phenotyping is a promising approach to further elucidate the impact of IL-6 blockade on immune-regulatory pathways. For monitoring of leukocyte (sub-)populations we will use reproducible immune monitoring panels for phenotyping. Recently, the international “The ONE study” consortium has designed a standardized panel (DuraClone®, Beckman Coulter, Marseille, France) for flow cytometry-based immune phenotyping that demonstrated robust results [29]. In the DuraClone immune monitoring kits, pre-defined assay tubes contain a layer with the dried-down antibody panel ready to use. Up to 10 different monoclonal antibodies per tube allows for the identification of leukocyte (e.g. T cell, B cell, natural killer cells) subpopulations present in whole blood samples.

#### Gene expression analysis

For gene expression analysis, 5 mL of blood will be collected in PAXgene Blood RNA tubes and stored at – 80 °C until analysis. After thawing, RNA will be isolated and transcribed into complementary DNA (cDNA). The amount of RNA/cDNA of IL-6 or IL-6R will be determined by quantitative real-time polymerase chain reaction (PCR) using the TaqMan assay.

#### Torque Teno virus (TTV) quantification

TTV DNA will be quantified in stored plasma samples using real-time PCR, as earlier described [30].

#### Pantoprazole PK

Liquid chromatography tandem mass spectrometry will be applied to assess pantoprazole levels and kinetics [27].

### Statistical methods

Analyses will be conducted according to the intention-to-treat principle. Continuous data will be presented as mean and standard deviation (SD) or median and interquartile range (IQR), as appropriate, and categorical variables as absolute and relative frequencies. Group comparisons (at baseline and after three months) for continuous data (CRP levels, eGFR, protein/creatinine ratio, levels of rejection biomarkers in serum and urine, IL-6/IL-6R expression levels, HLA antibody characteristics [DSA-MFI, number of DSA, virtual panel reactivity], morphological [e.g. g + ptc score, IF/TA score], or molecular (e.g. ABMR score) biopsy scores) will be analyzed using parametric (t test) or non-parametric (Mann–Whitney U test) tests, as appropriate. Fisher’s exact test will be used to compare categorical data between groups (occurrence of AEs, morphological/molecular ABMR categories, and biopsy-proven acute rejection at baseline and after three months). Transplant and patient survival or AE-free survival will be evaluated using Kaplan–Meier analysis and the Mantel Cox Log-rank test will be applied for group comparisons. For paired data (e.g. morphological and molecular biopsy scores after 3 vs 12 months in the overall cohort), paired t test or Wilcoxon test will be used. A two-sided *P* value < 0.05 will be considered statistically significant. Analysis of PK of clazakizumab and pantoprazol will include a description of the time evolution of antibody/drug concentration. Elimination half-life, Tmax, Cmax, clearance, and volume of distribution will be computed using standard software. For statistical analysis, IBM SPSS Statistics 24 (IBM Corporation, Armonk, NY, USA) and SAS for Windows (The SAS Institute Inc., Cary, NC, USA) will be used.

### Study registration

The study was approved by the Austrian (Federal Office for Safety in Health Care, Austrian Agency for Health and Food Safety) and German regulatory authorities (Federal Institute for Vaccines and Biomedicines, Paul-Ehrlich Institute). The study was registered in the European Clinical Trials Database (EudraCT number: 2017–001604-30; prospective registration) and in a public clinical trial database (ClinicalTrials.gov NCT03444103; retrospective registration).

## Discussion

Treatment of late/chronic ABMR after organ transplantation is a major challenge in clinical practice. This is underscored by the disappointing results of recent RCTs. In a double-blind placebo-controlled phase 2 trial, treatment of 44 kidney allograft recipients with two cycles of the proteasome inhibitor bortezomib failed to halt the progression of ABMR; there was no significant effect on the slope of eGFR, the morphological and molecular phenotype of rejection, and the levels of circulating DSA, respectively [17]. Moreover, in a multicenter trial (25 renal allograft recipients with chronic ABMR), the combined administration of CD20 antibody rituximab and intravenous immunoglobulin did not impact on the course of kidney function, DSA levels, or allograft morphology [16]. Finally, a small placebo-controlled pilot trial (15 patients) revealed that a six-month course of terminal complement inhibition using the anti-C5 antibody eculizumab had no effect on the results of follow-up biopsies, including the expression of endothelial cell-associated transcripts, with an at best marginal effect on the course of eGFR [31]. These data strongly reinforce that there is a great need for new innovative concepts for the treatment of late (chronic) ABMR.

IL-6 may be a promising target to interfere with ABMR. This pleiotropic cytokine plays a central role in inflammatory processes, guides the activation and differentiation of B cells, and plays an important role in the development of antibody-producing plasma cells. In a recent uncontrolled observational study, Choi et al. [22] reported on 36 kidney transplant recipients who received long-term treatment with the anti-IL-6R monoclonal antibody tocilizumab. They reported favorable graft survival, a decrease in the levels of circulating DSA, and a considerable reduction of microcirculation inflammation in follow-up biopsies. Importantly, IL-6R blockade was reported to be safe and well tolerated in this patient cohort [22].

These data are promising and prompted us to plan a clinical trial designed to systematically evaluate the concept of antagonizing IL-6/IL-6R signaling in transplant rejection. The primary aim of our trial is to evaluate the safety and tolerability of humanized monoclonal anti-IL-6 antibody clazakizumab, the most potent and longest acting agent in the IL-6/IL-6R blocking category, in a cohort of transplant recipients diagnosed with late ABMR.

Until now, no data obtained in transplant patients on dual or triple immunosuppression are available, so that safety assessment remains a major objective, though preliminary data obtained with the IL-6R antibody tocilizumab in transplant recipients point to a beneficial safety profile of targeting of IL-6/IL-6R also in patients on calcineurin inhibitor-based immunosuppression [22]. For our trial we have chosen a low dose (25 mg) and a four-week dosing interval. For this dose, earlier trials performed in autoimmune disease have shown a beneficial safety profile and higher doses were not associated with superior response rates [20, 25].

From previous studies, results obtained in > 1000 clazakizumab-treated patients/participants are available for assessment of safety patterns. Overall, AEs reported for clazakizumab treatment are consistent with IL-6 blockade. In studies performed in rheumatoid arthritis or psoriatic arthritis [20, 24, 25], clazakizumab was administered at doses up to 320 mg; many patients received this antibody as an add-on to treatment with methotrexate. Reported AEs caused by clazakizumab were abnormalities in liver function parameters, commonly mild to moderate in severity (< 3× upper limit of normal [ULN] in most cases) and more frequent under concomitant methotrexate treatment. Clinically significant increases in liver function parameters were shown to occur only in a few patients. In addition, clazakizumab was shown to decrease neutrophil counts, whereby the majority of observed cases were mild/moderate in severity, without clinical sequelae. In phase 2 studies evaluating clazakizumab in arthritis, reported infection rates under repeated administration were low and comparable to patients allocated to control groups [20, 24, 25]. Nevertheless, intensified immunosuppression, in addition to standard maintenance immunosuppression, can be expected to be associated with an increased infection risk. Hence, a careful patient follow-up will include a close monitoring for infectious complications (bacterial, fungal, and viral infections, including serial measurement of BK, JC, and cytomegalovirus viremia in peripheral blood), including two interim analyses evaluated by the DSMB. In parallel, the effect of clazakizumab on the overall load of immunosuppression will be monitored evaluating TTV load, which was earlier found to be associated with infection risk (and at the same time rejection) [30, 32].

In trials of clazakizumab in arthritis, no case of gastrointestinal perforation was reported. However, given the low, but significantly increased risk of gastrointestinal perforation in other large studies evaluating IL-6/IL-6R blockers in rheumatologic diseases, such as tocilizumab [33], patients with a history of gastrointestinal perforation, diverticulitis, or inflammatory bowel disease will not be included in the present trial.

The two-step design of the trial will allow us to obtain first results on the efficacy of IL-6 blockade. Outcome measures in this respect will include an evaluation of leukocyte (sub-)populations in peripheral blood, markers of inflammation and endothelial cell activation/injury, and two sequential follow-up biopsies to dissect the impact of treatment on ABMR morphology and rejection-related gene expression patterns. In a first part of the trial, patients will be enrolled in a double-blind RCT. After three months, however, all participants will be subjected to treatment with clazakizumab, until the end of the study after 12 months. There are several reasons for our decision to offer treatment to all participants. In a recent uncontrolled preliminary observational study, interference with IL-6/IL-6R blockade in ABMR patients using tocilizumab was reported to be safe and well tolerated, and first results have suggested stabilization of kidney function in many of the treated patients [22]. In the light of these results, long-term administration of placebo in patients with ongoing ABMR, even under adjustment of baseline immunosuppression, may be ethically problematic. Moreover, we believe that two subsequent protocol biopsies planned in our trial, which are necessary for an in-depth analysis of the early and late effects of IL-6 blockade, would not be justified for long-term placebo treatment.

Our trial will not include patients with severe allograft dysfunction or acute deterioration of graft function; recipients with an eGFR < 30 mL/min/1.73 m^2^ and/or high levels of protein excretion (protein/creatinine ratio > 3500 mg/g) will not be enrolled. A marked impairment of kidney function may reflect irreversible chronic damage; for patients with advanced graft injury, a sustainable treatment benefit can no longer be expected.

Our trial includes a detailed evaluation of PK (clazakizumab concentration) and PD (suppression of CRP). Prior studies have shown that the half-life (about 30 days) of clazakizumab at different doses (30–640 mg) was very similar in healthy male individuals and in those with RA and was consistent with that expected for a humanized IgG1 antibody (unpublished data). Another important point is that blockade of the pleiotropic cytokine IL-6 may potentially interfere with CYP metabolism and thus may potentially affect the half-life of CYP-metabolized drugs [34]. To address this relevant issue, our protocol will include studies to evaluate the impact of IL-6 blockade on the PK of the CYP2C19 substrate pantoprazole.

To avoid imbalances between study groups, randomization will be stratified for study site, to avoid any center bias, and the type of ABMR (active ABMR without chronic lesions in the microcirculation versus chronic active ABMR). Stratification for renal morphology was chosen to account for the expected variability regarding the extent of chronic injury, most prominently, transplant glomerulopathy. One may argue that the presence of advanced irreversible chronic injury may substantially influence the responsiveness to specific treatment.

As in a recent trial evaluating the effect of proteasome inhibition in late ABMR [17], our planned study includes an increase in the level of baseline immunosuppression (triple therapy in all included participants; increased calcineurin inhibitor through levels). It is well established that underimmunosuppression, frequently a result of medication non-adherence, is a major trigger of DSA and ABMR, particularly in the context of high levels of tissue incompatibility [1, 35, 36]. One may speculate that insufficient levels of baseline immunosuppression promote the progression of ongoing ABMR, especially in the context of placebo treatment.

A limitation of this pilot trial is the small sample size and short follow-up period that may be too short to demonstrate meaningful differences with respect to clinical outcome parameters. In a recent study evaluating the effect of bortezomib on kidney function in patients with late ABMR, the sample size calculation based on historical data obtained in ABMR patients revealed a sample size of 44 recipients to detect a 5 mL/min/1.73 m^2^ difference in eGFR slope evaluated over 24 months [17]. Of course, eGFR slope is at best a surrogate of long-term allograft survival [37, 38] and a study evaluating the effect of a given treatment on transplant survival would require a substantial extension of follow-up and a large number of individuals to be included.

In the study by Choi et al. [22], an important observation was that four recipients in whom tocilizumab was prematurely stopped lost their allografts. This was discussed to be the result of a rebound effect by accumulated IL-6. One may speculate that rebound effects may be less relevant for direct IL-6 blockade, e.g. using clazakizumab. Nevertheless, after the final study visit (week 52) all study participants will be monitored for any sign of worsening of renal transplant function in regular follow-up visits for five months after the last dose (5× half-life) in outpatient clinics.

If our planned study demonstrates that clazakizumab treatment is safe and well tolerated and if first results point to favorable immunological effects or even an influence on DSA levels or ABMR progression, our results would provide a valuable basis for the design of a subsequent large trial to clarify the effect of this compound on major clinical endpoints. Such a definitive trial, which may also be designed to define the optimal dose and dosing interval, will be a particular challenge, given the large samples size and the extended follow-up needed to demonstrate meaningful effects on graft survival (or surrogate endpoints, such as eGFR decline or the results of late follow-up biopsies performed beyond 12 months after study initiation). In contrast to our planned study, such a trial will require the inclusion of a control group subjected to prolonged placebo treatment, which, in absence of any evidence-proven treatment, may be ethically justifiable.

## Trial status

Recruitment of study patients started in January 2018. We expect completion of the trial in August 2020.

## Additional file

Additional file 1: SPIRIT 2013 checklist: Recommended items to address in a clinical trial protocol and related documents. (PDF 1888 kb)

## Abbreviations

ABMR: Antibody-mediated rejection
AE: Adverse event
ALT: Alanine aminotransferase
AST: Aspartate aminotransferase
CKD-EPI: Chronic Kidney Disease Epidemiology Collaboration
CRP: C-reactive protein
CYP: Cytochrome
DSA: Donor-specific antibodies
DSMB: Data and safety monitoring board
eGFR: Estimated glomerular filtration rate
GCP: Good Clinical Practice
GLP: Good Laboratory Practice
HLA: Human leukocyte antigen
IL-6: Interleukin-6
IL-6R: Interleukin-6 receptor
MFI: Mean fluorescence intensity
MMF: Mycophenolate mofetil
mTOR: Mammalian target of rapamycin
PD: Pharmacodynamics
PK: Pharmacokinetics
SAE: Serious adverse event
ULN: Upper limit of normal
